# Real‐Time MRI With Deep Learning for Efficient Evaluation of Neuromuscular Breathing Impairment

**DOI:** 10.1002/mco2.70579

**Published:** 2026-02-24

**Authors:** Rachel Zeng, Omar Al‐Bourini, Leonie Lettermann, Leon Lettermann, Ulrike Olgemöller, Sabine Hofer, Matthias Boentert, Tim Friede, Manuel Nietert, Dirk Voit, Jens Frahm, Martin Uecker, Ali Seif Amir Hosseini, Jens Schmidt

**Affiliations:** ^1^ Department of Neurology University Medical Center Göttingen Göttingen Germany; ^2^ Department of Clinical and Interventional Radiology University Medical Center Göttingen Göttingen Germany; ^3^ Institute for Theoretical Physics Heidelberg University Heidelberg Germany; ^4^ BioQuant Heidelberg University Heidelberg Germany; ^5^ Department of Cardiology and Pneumology University Medical Center Göttingen Göttingen Germany; ^6^ Neuromedical Centre, Department of Sleep Medicine Klinikum Osnabrück Osnabrück Germany; ^7^ Münster University Münster Germany; ^8^ Department of Medical Statistics University Medical Center Göttingen Göttingen Germany; ^9^ Department of Medical Bioinformatics University Medical Center Göttingen Göttingen Germany; ^10^ Biomedical NMR Max Planck Institute For Multidisciplinary Sciences Göttingen Germany; ^11^ Institute of Biomedical Imaging Graz University of Technology Graz Austria; ^12^ Else Kröner Fresenius Center for Optogenetic Therapies University Medical Center Göttingen Göttingen Germany; ^13^ Department of Neurology and Pain Treatment Immanuel University Hospital Rüdersdorf, Brandenburg Medical School Theodor Fontane Rüdersdorf Germany; ^14^ Faculty of Health Sciences Brandenburg Brandenburg Medical School Theodor Fontane Rüdersdorf Germany

**Keywords:** breathing pattern, convolutional neural network, diaphragm, dynamic imaging, quantitative MRI, respiratory muscle weakness

## Abstract

Efficient detection of breathing impairment is critical for treatment and prognosis in neuromuscular disorders. However, standard pulmonary function tests often yield ambiguous results. This prospective study evaluates whether advanced real‐time MRI (RT‐MRI) combined with deep learning‐based image segmentation provides sensitive outcome measures for respiratory dysfunction in late‐onset Pompe disease (LOPD), a model disease for diaphragmatic weakness. Eleven Pompe patients (mean age 52.2 years; 55% female) and 11 controls (mean age 50.9 years; 55% female) were included. RT‐MRI with a temporal resolution of 50 ms, combined with U‐Net‐supported lung segmentation, revealed significantly reduced diaphragmatic motion in Pompe patients compared to controls and unmasked paradoxical diaphragmatic motion in Pompe patients (7 of 11). Reduced diaphragmatic sniff velocity and pathological diaphragmatic/thoracic synchronicity were detected in Pompe patients with still normal results in standard pulmonary function tests. Fatty involution of the diaphragm as quantified by fast T1 mapping correlated significantly with functional parameters from RT‐MRI and pulmonary function tests. RT‐MRI combined with deep learning‐based lung segmentation offers novel biomarkers for early detection of respiratory muscle weakness. This new technique provides useful outcome measures for clinical care as well as treatment studies in patients with neuromuscular breathing impairment. The technique can also be used to characterize physiologic breathing patterns in healthy individuals.

## Graphical abstract

Breathing impairment is an important and common symptom in neuromuscular disorders. Reliable diagnostics are highly warranted. Real‐time MRI and deep learning‐based lung segmentation were used to analyze respiratory mechanics in patients with Pompe disease, a myopathy with diaphragmatic weakness, compared to healthy controls. Time‐resolved analysis enabled precise quantification of pathological breathing patterns and identified new, sensitive biomarkers for respiratory impairment.

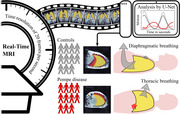

## 1 Introduction

Neuromuscular disorders (NMD) include a wide range of neurological diseases affecting skeletal muscles, peripheral nerves, or the neuromuscular junction. In these disorders, respiratory muscle weakness is frequently observed and substantially adds to both morbidity and mortality. Respiratory impairment may rapidly occur in Guillain–Barré syndrome or myasthenic crisis or gradually evolve in motor neuron disease or hereditary myopathies such as Pompe disease. In a disease continuum, weakness of the inspiratory muscles leads to sleep‐related hypoventilation, exercise intolerance, pulmonary infections, and premature death [1]. The diaphragm is the most important muscle for inspiration and its dysfunction is often the cause of respiratory insufficiency in NMDs [2]. Non‐invasive ventilation (NIV) has been shown to improve overall prognosis in various NMD with respiratory muscle involvement [3]. Therefore, early detection of breathing impairment is crucial for initiating supportive treatment. However, due to reduced exercise capacity, patients may not complain of dyspnea until a substantial respiratory muscle weakness has occurred. Furthermore, early signs of respiratory muscle weakness may be masked by compensatory breathing activities, for example, moderate diaphragmatic weakness can be compensated by increased movement of accessory inspiratory muscle. Standard pulmonary function tests (PFTs) are the primary diagnostic tool to detect and monitor respiratory muscle involvement, but consists of volitional tests and may show significant variability [4, 5]. Overnight transcutaneous capnometry is used to early detect alveolar hypoventilation [6], but often requires hospitalization and may not be easily accessible. Other non‐volitional tests include phrenic nerve conduction studies and magnetic stimulation of the diaphragm, combined with invasive measurement of the transdiaphragmatic pressure [7, 8]. However, these procedures bear methodological limitations or cannot be considered suitable for routine clinical practice. In addition to the clinical importance of sensitive diagnostic tools, robust outcome measures for respiratory muscle function are needed for the growing number of clinical trials in NMDs, driven by scientific advancements in therapies like gene therapy and antisense oligonucleotides in recent years.

Several imaging modalities have emerged to assess diaphragmatic function, including ultrasound [9], fluoroscopy [10], CT [11], and MRI [12, 13, 14, 15, 16, 17], with MRI offering important advantages such as radiation‐free imaging and the potential to study distinct respiratory muscles. In order to study complex respiratory movements during free breathing, dynamic MRI with adequate spatial resolution and high‐speed image acquisition is desirable. Advanced real‐time MRI (RT‐MRI) using highly undersampled radial fast low‐angle shot (FLASH) image acquisition [18] with non‐linear inverse reconstruction [19, 20] offers continuous acquisition of high‐quality images with unprecedented temporal resolution. The RT‐MRI technique also allows for fast T1 mapping (T1FLASH) [21], through which T1 values of specific tissues can be calculated. It has been used to characterize myocardial tissue [22] and to quantify degenerative changes in skeletal muscle [23]. For efficient analysis of large data volumes generated by MRI, deep learning‐based image segmentation is increasingly used. U‐Net is a specific convolutional neural network (CNN), a type of deep‐learning algorithm, that has been applied to various biomedical image segmentation, with its use and potential still growing [24, 25].

Here, we demonstrate the use of novel real‐time MRI (RT‐MRI) at 50 ms temporal resolution combined with U‐Net supported lung segmentation to study respiratory involvement, as well as the application of fast T1 mapping to assess fatty involution of the diaphragm in patients with late‐onset Pompe disease (LOPD). LOPD was chosen as a model disease for respiratory involvement in NMD because it is a slowly progressive myopathy with predominant involvement of the diaphragm in early stages of the disease [26]. The aim of this study was to evaluate whether RT‐MRI provides more sensitive indicators of respiratory dysfunction compared to PFTs and diaphragm ultrasound and to gain new insights into disease‐specific pathomechanisms of respiratory muscle weakness.

## 2 Results

### 2.1 Patient Characteristics, PFTs, and Ultrasound of the Diaphragm

Eleven patients with LOPD (age 52 ± 13 years, five female) and 11 control subjects (age 51 ± 15 years, five female) were enrolled. Five LOPD patients received non‐invasive ventilatory support at night. Table 1 shows the mean values of the clinical characteristics, as well as the results of PFTs and diaphragm ultrasounds; individual results for each study participant can be obtained from Tables S1 and S2. PFTs revealed significantly lower FVC and VC in LOPD patients compared to the controls, especially pronounced in supine position. Blood gas analysis showed a significantly higher pCO_2_ in LOPD patients, although still within the physiological range. Diaphragm ultrasound showed a significantly reduced mobility of the diaphragm during deep breathing and sniff maneuver in LOPD patients compared to the controls.

**TABLE 1:** Clinical characteristics of the study cohort and results of pulmonary function tests, blood gases, and ultrasound assessment of diaphragmatic motion. As there was no significant difference in ultrasound measurements of the right and left hemidiaphragm, only results from the right hemidiaphragm are depicted.

Patient baseline characteristics	Control (*n* = 11) Mean (SD) [range]	Pompe (*n* = 11) Mean (SD) [range]	*p*‐value
Sex, F:M	5:6 ratio	5:6 ratio	>0.99
Age at inclusion	50.9 (15.2) [24–77]	52.2 (12.9) [23–71]	0.687
Age of onset	n.a.	36.8 (14.6) [13–56]	n.a.
BMI	26.2 (4.36) [20–33]	22.6 (2.69) [20–27]	0.056
MRC muscle sum score	138 (3) [133–140]	132 (9) [108–139]	**<0.001**
mMRC dyspnoe scale	0.273 (0.467) [0–1]	1 (0.623) [0–2]	**0.017**
R‐PAct‐score	n.a.	24.1 (5.13) [16–31]	n.a.
ERT at time of study	n.a.	Yes = 9	n.a.
Ventilation therapy	n.a.	4 BIPAP, 1 CPAP (at night)	n.a.
Wheelchair use	0	1	>0.99
**Pulmonary function tests/blood gases**			
FVC upright (% of reference)	92.7 (10.4) [78–108]	74.1 (12.1) [56–96]	**0** **.002**
FVC supine (% of reference)	90.3 (9.4) [73–105]	57.8 (12.1) [42–82]	**<0.001**
MIP (kPa)	7.1 (3.1) [2.7–12.4]	4.9 (2.5) [0.65–9.6]	0.13
pCO2 (mmHg)	35.1 (2.21) [33–38]	39.3 (3.43) [35–45]	**0.006**
pO2 (mmHg)	82.3 (9.72) [67–102]	73.8 (8.38) [60–89]	0.075
**Diaphragmatic excursion on ultrasound (mm)**			
Tidal breathing seated	23.4 (7.9) [13.1–35.4]	16.5 (5.2) [6.7–23.8]	**0.038**
Tidal breathing supine	19.3 (6.1) [7–28.7]	15.6 (4.5) [10.6–23.2]	0.151
Deep breathing seated	44.7 (12.2) [28.6–65.2]	24.4 (6.57) [15.6–36.7]	**<0.001**
Deep breathing supine	41.9 (11.1) [26.4–65.7]	23.1 (7.1) [12.4–36.4]	**<0.001**
Sniff seated	39.6 (10.5) [21.2–56]	21.8 (7.3) [12.3–34.2]	**<0.001**
Sniff supine	39.3 (14.1) [22.4–64.4]	21.4 (7.8) [9.8–36.2]	**<0.001**

*Note: p*‐values indicate differences between the two groups and were calculated using the Mann–Whitney *U* analysis for continuous data and Fisher's exact test for categorical data. *p* ≤ 0.05 is considered statistically significant and indicated in bold.

Abbreviations: BIPAP, Biphasic Positive Airway Pressure; BMI, body mass index; CPAP, continuous positive airway pressure; ERT, enzyme replacement therapy; FVC, forced vital capacity; MIP, maximum inspiratory pressure; MRC, Medical Research Council; R‐PAct, Rasch‐built Pompe‐specific activity; n.a., not applicable; SD, standard deviation.

### 2.2 Development of Outcome Measures for RT‐MRI and Automatic Lung Segmentation

**FIGURE 1:**
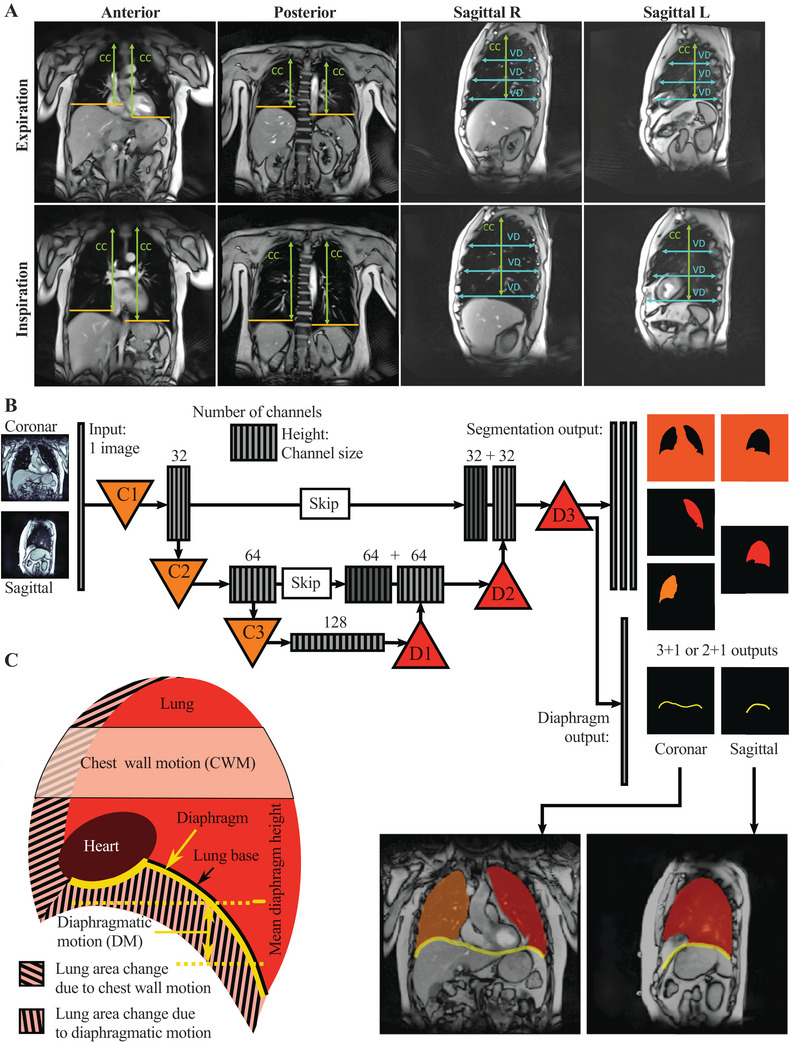
Overview of the manual and automatic analysis and definition of key quantities. (A) Exemplary MRI images in expiration (top row) and inspiration (bottom row) representing the four different imaging planes used for manual analysis: A coronal slice of the anterior plane, showing the left ventricle as the anatomical lead structure, a coronal slice of the posterior plane, showing the spine as anatomical lead structure, and sagittal slices of the left and right lung, respectively. Manual analysis encompassed the relative change between inspiration and expiration of the craniocaudal height of the lung (CC) and the ventrodorsal excursion of the chest wall (VD). (B) Schematic depiction of the U‐Net architecture employed for automatically segmenting lung and diaphragm configuration. A single input image, either coronal or sagittal, is transformed by three convolutional blocks (C1–C3) into many of the same image, with lower resolution representations, highlighting different features in each channel. Subsequent deconvolution (D1‐D3) regains the original size, generating segmentation into “no‐lung”, "right lung", "left lung" (coronal) or “no‐lung” and "lung" (sagittal). As an additional output, the diaphragm configuration is inferred. (C) Definition of relevant outcome measures: Important aspects of the lung (red) are the area over which the chest wall motion (CWM) averaged, as well as the lung base (black) and the diaphragm (yellow). Yellow dashed lines give the averaged diaphragm height, the difference of which is the diaphragmatic motion (DM). Shaded areas (left and right) illustrate thoracic lung area change, lower shaded areas the caudal change of lung area through the diaphragm. From these areas, the respective proportions of area change (PC) relative to total lung area change are obtained, PC chest wall and PC diaphragm, respectively.

A major challenge of this study was to develop an analysis pipeline for respiratory motion features that could utilize the large amount of data provided by RT‐MRI, which resolves 10 s of respiratory motion with 200 images per MRI slice position. For evaluation of coronal sequences, two imaging planes were selected: an anterior plane identified by the left ventricle and a posterior plane identified by the spine and/or descending aorta. From the sagittal sequences, one plane was selected from each of the right and left lungs for further analysis (Figure 1A).

For manual analysis, the imaging planes showing maximal inspiration and expiration were identified (Figure 1A). Diaphragmatic excursion was determined by the *craniocaudal (CC) height of the lung*, while thoracic motion was measured by the *ventrodorsal (VD) excursion of the chest wall* at lung base, one‐third and two‐third of lung height (Figure 1A). All outcomes were calculated as relative change (Δrel) between maximal inspiration and expiration [(length_insp_ − length_exp_)/length _exp_] (Figure 1A).

The automatic analysis pipeline consisted of a segmentation and an evaluation part. Segmentation was performed with a U‐Net [24], classifying the pixels in the MR images into “no‐lung” and “lung” for sagittal or “no‐lung”, “right lung”, and “left lung” for coronal planes, and additionally tracing the diaphragm configuration (Figure 1B). After training the network using input images and the desired output masks, the segmentation allows to extract different quantities characterizing respiratory motion (Figure 1C): From the individual diaphragm shape, a mean craniocaudal height was obtained, and the time series of mean heights yielded the *diaphragmatic motion* (*DM)*. *Chest wall motion (CWM)* was obtained by averaging over a height domain in the upper part of the lung and computing a mean width of the lung for each frame during the breathing cycle. The Pearson correlation coefficient between the time‐series of diaphragmatic and chest wall motion yielded a measure of *synchronicity* ranging from a value of +1 for a synchronous extension to a value of −1 for asynchronous extension of the lung. *Total lung area change (LA)* was computed as the maximal amplitude of the lung areas in the different imaging planes. To measure the separate contribution of the diaphragmatic and the chest wall motion to LA, the lines of contact between the diaphragm and lung base, and between the chest wall and lung, were determined. The areas swept by these lines were obtained via analytical integration for subsequent frames, yielding the mean *proportions of area change (PC)* (Figure 1C). A diaphragmatic PC of zero shows that none of the total lung area change during the breathing cycle was caused by diaphragmatic motion and, vice versa, a value close to 1 indicates that nearly all of it was caused by diaphragmatic motion. For the sniff maneuver, the maximum velocity reached by diaphragmatic motion (*diaphragmatic sniff velocity*) and chest wall motion (*thoracic sniff velocity*) were quantified. To avoid overestimation of the velocity due to positional noise, the relevant curves were smoothed over five frames using a Gaussian filter, and the velocity was measured by taking the difference over three frames.

For visual analysis, heatmaps were computed by color‐coding for each pixel on how many frames were occupied by lung tissue. Time dependent quantities were visualized as graphs. Furthermore, the segmentation and important quantities were exported as videos (Movies S1–S5) for verification of correct segmentation.

### 2.3 Motion Analysis of Respiratory Muscles Using RT‐MRI

**FIGURE 2:**
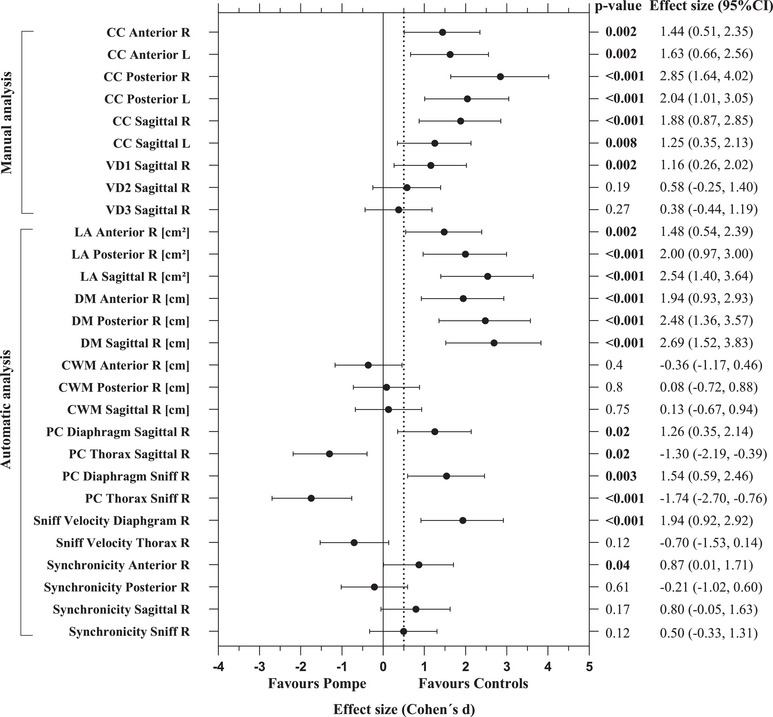
Main outcome measures from RT‐MRI assessment of diaphragmatic and chest wall motion in patients with late‐onset Pompe disease and controls. Forest plot of Cohen's *d* effect sizes and corresponding 95% confidence intervals (CI) for RT‐MRI outcomes; for better overview, only results from the right hemithorax are shown for the automated analysis. A Cohen's *d* of 0.5 is considered a medium effect size (dotted line) and 0.8 is considered a large effect size. Significance is reported as *p*‐values obtained by unpaired Mann–Whitney *U* test. Anterior, anterior imaging plane; CC, cranio‐caudal change of lung size; CWM, chest wall motion; DM, diaphragmatic motion; LA, total lung area change; PC, proportions of area change relative to total lung area change; Posterior, posterior imaging plane; R, right hemithorax; Sagittal, sagittal imaging plane; VD, ventrodorsal change of lung size.

Evaluation of the RT‐MRI data revealed several significant differences in respiratory motions between LOPD patients and controls. To compare the efficacy of the different MRI outcome measures for detecting differences between LOPD and controls, effect sizes were calculated (Cohen's *d*) and the corresponding forest plot is shown in Figure 2. Respective scatter plots of all MRI outcomes are provided in Figures S1 and S2; ROC curves displaying the diagnostic performance of selected outcomes are shown in Figure S3.

Manual analysis of the craniocaudal relative change of lung size during deep breathing showed a decreased mobility of the diaphragm in LOPD patients compared to controls, which could be observed in all analyzed imaging planes and was most prominent in the posterior coronal plane (*p* < 0.001) (Figure S1). In LOPD patients, the right hemidiaphragm was more affected than the left side, while the control group showed a relatively similar motion range of both hemidiaphragms. Chest wall motion as measured by the ventrodorsal relative change of lung size was slightly decreased in the apical part of the lung in LOPD patients compared to controls (*p* = 0.002) (Figure S1).

**FIGURE 3:**
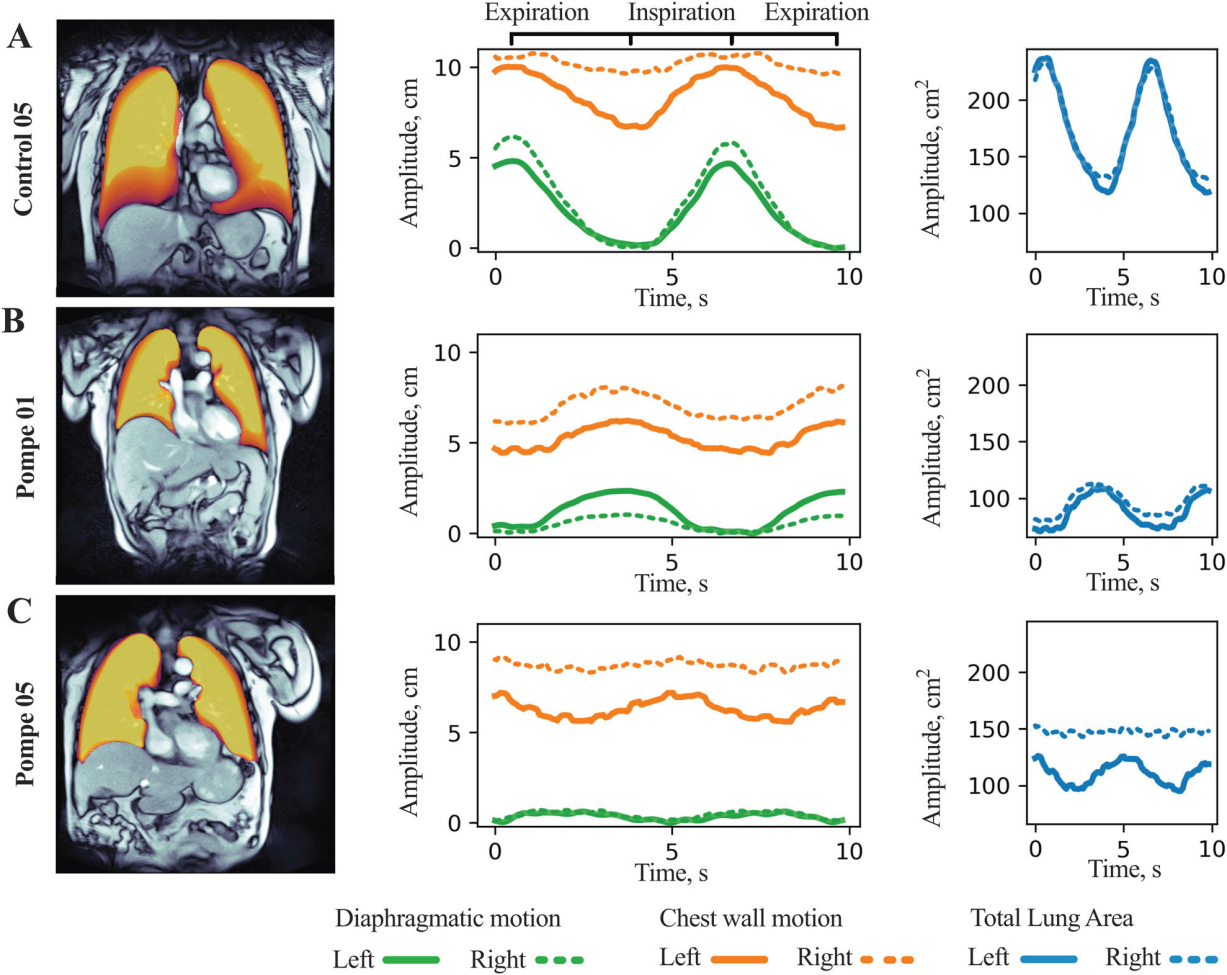
RT‐MRI assessment of diaphragmatic and chest wall motion in patients with late‐onset Pompe disease and controls. (A and B) Exemplary MRI images of a control subject and a Pompe patient in the anterior coronal plane. The movement patterns of the lung area are visualized as heatmaps, bright yellow depicts the area continuously occupied by lung tissue, whereas dark orange visualizes the area to which the lungs additionally extend during deep inspiration. The time series in the middle panels show the dynamics of the diaphragmatic motion (green) and the chest wall motion (orange), while the right panels show total lung area displacement (blue), illustrating significantly reduced diaphragmatic motion and lung area change for the Pompe patient. (C) Example of a Pompe patient with asynchronous breathing pattern, that is, the diaphragm is moving upwards while the chest wall is extending.

Using the U‐Net‐based automatic lung segmentation, motion profiles of the diaphragm and chest wall could be obtained over the entire time series of deep breathing, as shown in Figure 3 and Movies S1–S3, further visualizing the decreased DM in LOPD patients (*p* < 0.001). CWM showed no significant differences when compared to controls in either the coronal or sagittal planes (*p* = 0.8 and *p* = 0.75). Quantification of the differential contribution of diaphragm and chest wall to total lung area change (*proportion of area change* [PC]) clearly demonstrated that the diaphragm is the main component of respiratory movement in the control group with PC_Diaphragm_ 0.69 ± 0.16 (mean ± SD) and PC_Thorax_ 0.25 ± 0.14, when analyzed for the right hemidiaphragm in the sagittal plane. In LOPD patients, the contribution of the chest wall was relatively larger due to the pronounced diaphragmatic weakness with PC_Diaphragm_ 0.35 ± 0.33 and PC_Thorax_ 0.58 ± 0.32 (both *p* = 0.02 compared to controls) (Figure 3A,B, Figure S2g, and Movies S1–S2).

Analysis of synchronicity between diaphragmatic and chest wall movements yielded values between +1 (complete synchronicity: diaphragm and chest wall expand and deflate at the same time) and −1 (complete asynchronicity: diaphragm and chest wall expand and deflate oppositely). Control subjects showed significantly higher synchronicity between diaphragmatic and chest wall movement (Figure 3A) with mean values of 0.89 ± 0.10 when analyzed for the right hemidiaphragm in the anterior‐coronal plane. In comparison, LOPD patients showed mean synchronicity values of 0.53 ± 0.60 in the same plane (*p* = 0.04), with four LOPD patients exhibiting below‐average results, including one patient with even negative value of synchronicity due to paradoxical diaphragmatic movement (Figure 3C, Figure S2e, and Movie S3).

**FIGURE 4:**
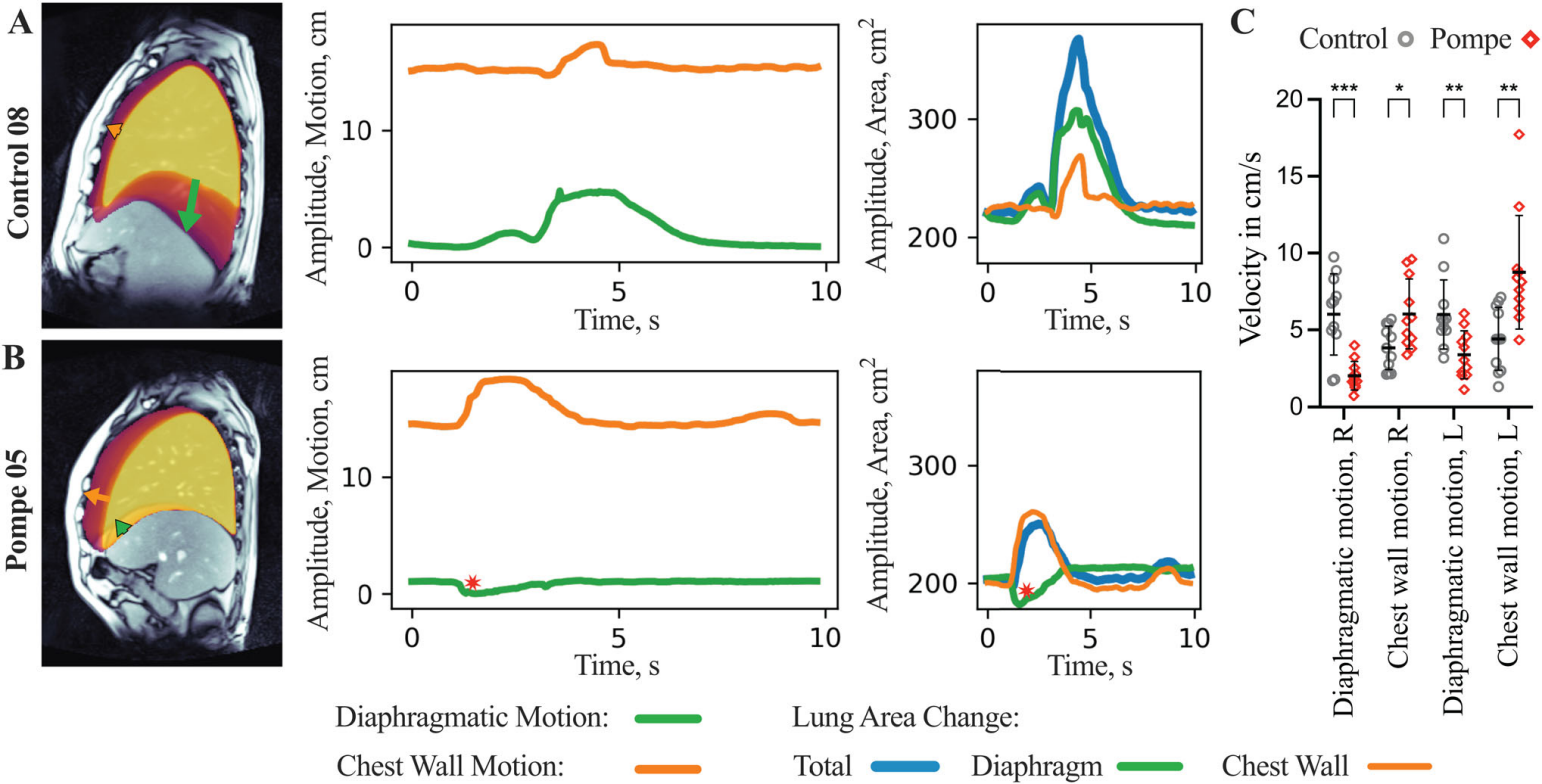
RT‐MRI assessment of respiratory movements during sniff maneuver unmasks paradoxical movement of the diaphragm in patients with late‐onset Pompe disease. (A and B) Exemplary MRI images of a control subject and Pompe patient in the sagittal plane of the right lung. The time series in the middle panels show the corresponding development of the diaphragmatic motion (green), the chest wall motion (orange), and the total lung area change (blue) with the respective area change contributed by diaphragmatic motion (green) and chest wall motion (orange). Note the paradoxical upward movement of the diaphragm in the Pompe patient shown in B) (middle panels, marked with a red asterisk). (C) Velocity of diaphragmatic motion and chest wall motion based on maximum lung extension during sniff maneuver for the right sagittal plane (R) and left sagittal plane (L).

The sniff maneuver unmasked a paradoxical diaphragmatic movement in seven out of 11 LOPD patients, but not in control subjects. Automatic analysis of motion pattern revealed that, while the sniff maneuver starts with a caudal displacement of the diaphragm in control subjects (Figure 4A and Movie S4), seven out of 11 LOPD patients showed a paradoxical cranial displacement of the diaphragm during initiation of the maneuver (Figure 4B and Movie S5). Furthermore, RT‐MRI allowed to quantify the velocity of either diaphragm and chest wall excursion during voluntary sniffing, termed sniff velocity. Compared to the control group, LOPD patients showed a significantly lower diaphragmatic sniff velocity (*p* < 0.001) and a trend toward a higher thoracic sniff velocity (*p* = 0.12) (Figure 4C). Interestingly, PC_Thorax_ in LOPD patients was larger with a voluntary sniff maneuver (*p* < 0.001) than with deep breathing (*p* = 0.02) (Figure S2g,i), reflecting additional rib cage expansion during sniffing to compensate for diaphragm weakness.

### 2.4 T1 Mapping of the Diaphragm

**FIGURE 5:**
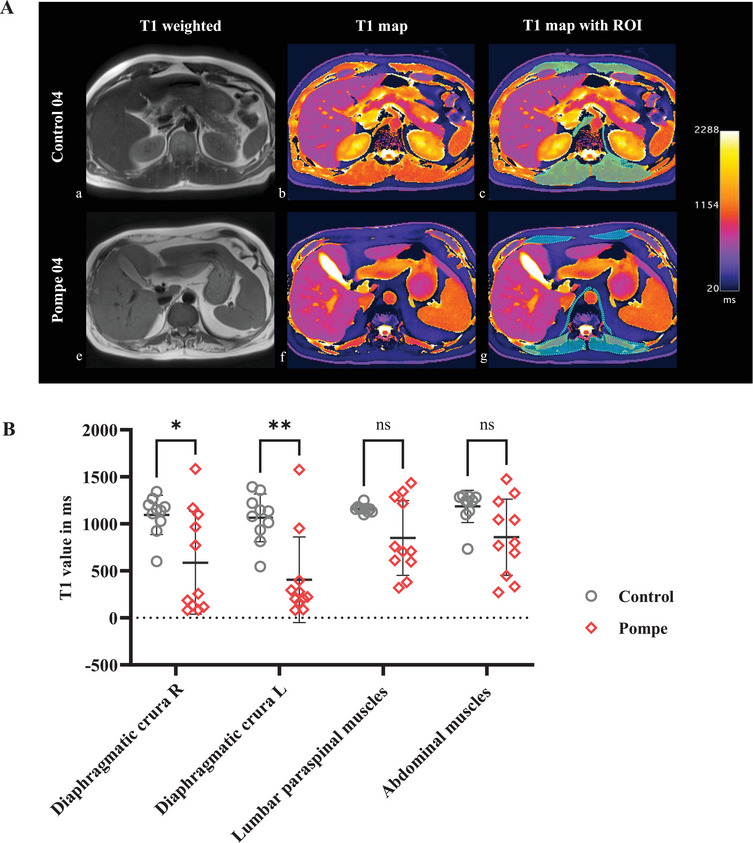
T1 mapping of diaphragmatic crura and trunk muscles in late‐onset Pompe disease versus control subjects. (A) Selected axial T1w images (a and e) and corresponding T1 maps (b, c, f, g) of the upper abdomen in a male healthy control and a male Pompe patient, both aged between 40 and 50 years. (c and g) show examples of how the regions of interest (ROI) are drawn to obtain mean T1 values (in ms) of the diaphragmatic crura, abdominal and paraspinal muscles. Note the severe atrophy and decreased T1 values of the analysed muscles in the Pompe patient. (B) T1 mapping reveals significantly lower T1 values of the right (R) and left (L) diaphragmatic crura in Pompe patients, indicating a higher intramuscular fat content. T1 mapping of the abdominal and lumbar paraspinal muscles (both calculated as mean values of the right and left side, respectively) showed a trend toward lower mean T1 values in Pompe patients when compared to controls, with a broader variation of T1 values in the Pompe cohort. Significance is reported as *p*‐values obtained by unpaired Mann–Whitney *U* test.

T1 mapping of the diaphragmatic crura revealed significantly lower mean T1 values in LOPD patients (right crus 586.11 ± 545.85 ms, left crus 405.31 ± 455.37 ms) compared to controls (right crus 1094.87 ± 208.94 ms, left crus 1064.38 ± 254.05 ms) (LOPD vs. controls: right crus, *p* = 0.02 and left crus, *p* = 0.002), indicating a higher fat content of the analyzed muscle (Figure 5). The extent of atrophy and fatty infiltration of the diaphragmatic crura varied significantly among LOPD patients, ranging from moderate involvement to near end‐stage fatty replacement of muscle tissue, as quantified by T1‐mapping.

Analysis of the abdominal and lumbar paraspinal muscles in the same field of view (FOV) as the diaphragmatic crura also showed a trend toward lower mean T1 values in LOPD patients (abdominal muscles 858.06 ± 405.05 ms; paraspinal muscles 851.02 ± 397.83 ms) when compared to controls (abdominal muscles 1185.16 ± 172.24 ms; paraspinal muscles 1157.85 ± 39.91 ms) (LOPD vs. controls: abdominal muscles, *p* = 0.28 and paraspinal muscles, *p* = 0.05) (Figure 5). The relative preservation of chest wall motion suggests a sparing of the intercostal muscles in Pompe disease, but the intercostal muscles were too small to be reliably assessed by T1 mapping. Individual results of T1 mapping of each study participant can be obtained from Table S3.

### 2.5 Correlation of MRI Findings With Diaphragm Ultrasound and PFTs

**FIGURE 6:**
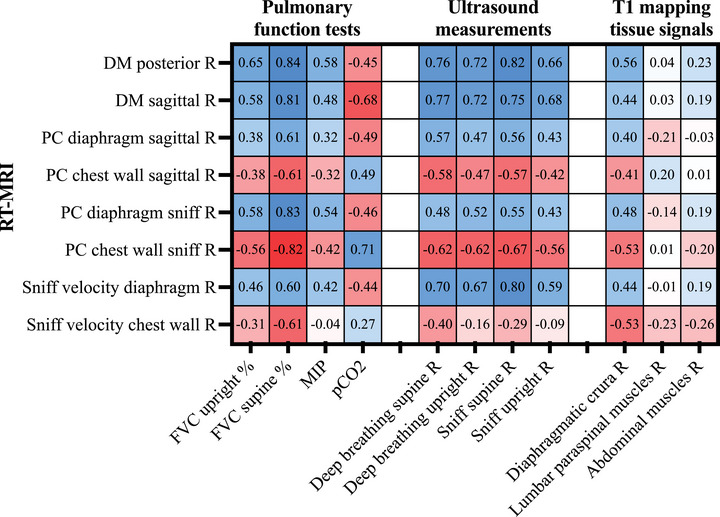
Correlation matrix of Spearman's correlation coefficients for the multimodal assessment of breathing in patients with late‐onset Pompe disease and controls. DM, diaphragmatic motion; FVC, forced vital capacity; L, left hemithorax; MIP, maximal inspiratoric pressure; PC, proportion of area change relative to total lung area change; R, right hemithorax.

We observed strong correlations (*r* > 0.6) between diaphragm‐related RT‐MRI outcomes with ultrasound of diaphragmatic movement, FVC % of predicted in supine position and to lesser extent with FVC % of predicted in seated position and MIP (Figure 6). Notably, blood gas pCO_2_ showed a significant negative correlation with diaphragm‐derived MRI outcome measures. Interestingly, contribution of chest wall to total lung area change as well as thoracic sniff velocity showed significant negative correlation with predicted FVC supine, indicating their role as compensatory mechanisms for diaphragm weakness. T1 values of the diaphragmatic crura correlated significantly with diaphragmatic motion parameters obtained by RT‐MRI (*r* ranging between 0.40 and 0.56) and predicted FVC seated and supine (*r* ranging between 0.61 and 0.69). T1 values of the abdominal muscles or lumbar paraspinal muscles did not show significant correlations with RT‐MRI outcomes or PFT. The full matrix of correlation coefficients is shown in Figure 6.

## 3 Discussion

We here demonstrate that advanced RT‐MRI in combination with U‐Net supported automatic lung segmentation was an effective tool for evaluating pathological breathing mechanisms and for characterizing new outcome measures associated with diaphragmatic weakness in a neuromuscular disorder.

Previous studies have used dynamic MRI to evaluate respiratory function in Pompe disease [13, 16, 17], demonstrating two key diagnostic advantages over PFTs and ultrasound: it allows simultaneous assessment of different respiratory muscles during breathing and enables detailed analysis of regional diaphragm motion. However, due to the lack of continuous, untriggered live acquisition, these studies have been limited in their ability to visualize pathological breathing patterns. Mogalle et al. detected paradoxical diaphragmatic movement in one out of 10 Pompe patients on a slow expiratory maneuver, while Harlaar et al. found no evidence of paradoxical movement in a cohort of 22 Pompe patients using dynamic MRI with a temporal resolution of 200 ms per frame (= 5 fps) [17], which is four times slower than the technique used in the present study. Our present study utilized RT‐MRI with a temporal resolution of 50 ms per frame (= 20 fps) and without the need for triggering, retrospective gating or breath holding, thus visualizing respiratory movements in true real time [27]. This allowed us to analyze sniff maneuver, a rapid respiratory maneuver that recruits maximal inspiratory effort, showing clear paradoxical diaphragmatic movement in seven out of 11 LOPD patients. Pathological breathing patterns were further revealed by analysis of diaphragmatic and thoracic sniff velocities, which differed significantly between controls and LOPD patients. Pathological respiratory motions during sniff maneuver have previously been visualized by ultrasound, fluoroscopy and dynamic chest radiography [28, 29]. In comparison, RT‐MRI offers the advantage of radiation‐free imaging at high spatial and temporal resolution, as well as simultaneous analysis of chest wall and diaphragmatic motion during breathing maneuvers.

Different strategies have previously been used to segment lung contours for kinematic analysis, including manual segmentation [17], and semi‐automated methods using frame‐by‐frame feature extraction [15] or registration‐based pipelines [13]. While these methods allow for analysis of various diaphragmatic and thoracic motion features, deep learning‐driven algorithms offer faster processing and improved capture of complex anatomical changes. In this study here, the combination of RT‐MRI with deep learning–based image segmentation enabled efficient analysis of large MRI datasets and maximized the diagnostic potential. This includes the quantification of diaphragmatic/thoracic synchronicity as a new outcome measure for studying breathing dynamics. While healthy controls show high synchronicity between diaphragm excursion and chest expansion, LOPD patients show less synchronicity or even asynchronous movements of diaphragm and chest, indicating pathological breathing. An important criterion for respiratory outcome measures is their ability to detect early signs of breathing impairment. In the study by Haarlar et al., dynamic chest MRI showed that, in Pompe patients with normal spirometry results (13 out of 35 patients, FVC supine < 80% predicted), the diaphragmatic motion is already reduced and the diaphragm shape is more curved during inspiration [17]. In our study cohort, one patient (P06) showed an FVC supine within normal range. This patient also displayed impaired diaphragmatic motion and, additionally, a reduced synchronicity and diaphragmatic sniff velocity. Similarly, in another patient (P10) with normal FVC measured in seated position and only slight diaphragm weakness as seen by RT‐MRI, synchronicity and diaphragmatic sniff velocity were clearly reduced below average. Therefore, we propose impaired diaphragmatic/thoracic synchronicity and diaphragmatic sniff velocity as early indicators of respiratory dysfunction. Compared to measuring diaphragmatic excursion alone, these new MRI outcomes could enhance diagnostic sensitivity and reliability by capturing more complex aspects of diaphragm dysfunction. Further investigations of patients in the early stages of the disease are needed to confirm this. Previous studies have also suggested diaphragm orientation and curvature as parameters of early diaphragmatic weakness [13, 17, 30]. However, the shape of the diaphragm is subject to physiological factors such as age, weight, and thoracic dimensions [31]. Therefore, analytical approaches based on dynamic motion characteristics rather than geometrical features may be more reliable for assessing diaphragmatic function.

Consistent with previous MRI studies of respiratory motion in Pompe disease [11, 12, 13, 16, 17, 30], we found prominent diaphragmatic weakness in our cohort of LOPD patients, particularly pronounced in the right hemidiaphragm and the posterior part of the diaphragm. Due to the more subtle pathophysiological changes, there is controversy among studies regarding whether chest wall mobility is affected in Pompe disease [12, 13, 16, 17]. Most of these studies measured chest wall excursion as the width of the lung in the anteroposterior direction. However, this approach is error‐prone since the lung width at a single height is subject to natural deformation of the lung contour during breathing. This challenge was overcome in the present study by combining RT‐MRI with automatic lung segmentation, which allowed us to calculate a mean lung width by averaging over an area in the lung, not a length, and analyze its change over a full cycle of breathing. This approach confirmed that chest wall motility did not differ significantly between LOPD patients and controls, which indicates relative preservation of intercostal muscle strength in Pompe disease.

In this study here, quantitative T1 mapping (T1FLASH) demonstrated significantly decreased mean T1 values, indicating fatty or fibrous infiltration in the diaphragmatic crura in the majority of LOPD patients, which also correlated significantly with decreased diaphragmatic motion and reduced FVC. These findings are in line with previous MRI studies using semiquantitative visual grading for muscle fat replacement in the diaphragm of Pompe patients [11, 32]. In comparison, T1 mapping provides observer‐independent, absolute metrics of tissue composition, aiding correlation analyses between morphology and function of skeletal muscles, including the diaphragm. In recent years, quantitative muscle imaging has emerged as a valuable tool for characterizing and monitoring disease activity in neuromuscular disorders [33]. However, chemical shift imaging such as Dixon imaging, which is often used to quantify fat fractions in muscle tissue, can be limited by motion artifacts, whereas the T1‐mapping technique used in this study requires only seconds of breath holding. Therefore, we propose T1 mapping as a promising biomarker for diagnosis and follow‐up assessments of respiratory muscle involvement.

A limitation of the present study is that the patient cohort was small and heterogeneous regarding severity of respiratory involvement. Nevertheless, due to the pathognomonic diaphragmatic weakness in Pompe disease, we were able to delineate multiple MRI outcomes for characterization of pathological breathing mechanisms. In addition to longitudinal studies of Pompe patients with normal or mildly impaired respiratory function at baseline, further investigations in a healthy population for reference values and in other diseases with more subtle respiratory involvement would help to solidify the diagnostic value of the RT‐MRI technique described here. Another limitation is that RT‐MRI and automatic segmentation algorithms are not yet widely available. Therefore, further standardization and automation of segmentation processes are needed before application in daily practice becomes feasible.

In conclusion, advanced RT‐MRI is a reliable, sensitive, and non‐invasive diagnostic tool for assessing breathing impairment and respiratory muscle involvement. By combining RT‐MRI with U‐Net‐based automatic lung segmentation, we here identified new outcome measures that may be suitable for detecting early respiratory impairment in neuromuscular disorders and other diseases. The data contribute to a better understanding of breathing mechanisms and may be relevant for designing outcome measures in clinical trials and defining long‐term monitoring parameters in neuromuscular care. Beyond this, the technique can be useful for detailed characterization of physiologic breathing patterns.

## 4 Methods

### 4.1 Study Population

Eleven adult patients with genetically confirmed late‐onset Pompe disease (LOPD) and 11 controls matched by age, sex, and BMI were included. LOPD patients were recruited from the neuromuscular outpatient clinics of the University Medical Center Göttingen and the University Hospital Münster. Control participants included two healthy volunteers and nine patients who were treated at the Department of Neurology, University Medical Center Göttingen at the time of the study, including five patients with minor strokes, three patients with multiple sclerosis, and one patient with benign paroxysmal positional vertigo. None of the control participants had a history of respiratory disease or a condition affecting the musculoskeletal system. None of the study participants had contraindications for a 30‐min MRI examination.

### 4.2 MRI Examinations

Participants received an RT‐MRI survey including different breathing maneuvers. RT‐MRI was conducted using a 3T MRI system (Magnetom Skyra 3T, Siemens Healthineers, Erlangen, Germany), with an RF‐spoiled radial FLASH sequence [18] enabling an acquisition time of 20 frames per second (fps) when combined with image reconstruction via regularized nonlinear inversion (NLINV) with temporal regularization to achieve high undersampling [19, 20].

This protocol contained RT‐MRI sequences of the thorax with consecutive T1‐weighted two‐dimensional images, each plane recorded for 10 s, to allow for an optimal spatial in‐plane (1.25 mm (sagittal), 1.5 mm (coronal)) and temporal resolution (20 fps or 50 ms per frame). Further sequence parameters for coronal images were as follows: TR 2.17 ms, TE 1.32 ms, slice thickness 8 mm, FOV 448 mm; for sagittal images: TR 2 ms, TE 1.21 ms, slice thickness 8 mm, FOV 448 mm. A stack of 7–15 coronal sequences spaced 2 cm apart were obtained covering the entire thorax during maximal deep breathing. Two sagittal planes were positioned through the apex of the lungs and the center of the hemidiaphragmatic domes, respectively. Sagittal images were obtained during maximal deep breathing and a sniff maneuver, where patients were instructed to inhale rapidly through the nose. With all study participants, voluntary respiratory maneuvers were thoroughly practiced prior to image acquisition.

Additionally, quantitative T1 mapping was performed for assessment of fatty infiltration of the diaphragmatic crura. The technique used here is based on a single‐shot inversion‐recovery method with serial acquisitions of highly undersampled radial FLASH images and iterative reconstruction in time‐reversed order using NLINV followed by pixel‐wise fitting of the T1 relaxation curve [21]. Sequence parameters were as follows: TR 2.38 ms, TE 1.47 ms, slice thickness 6 mm, FOV 320 mm, and spatial in‐plane resolution 1.25 mm. T1 maps were obtained as axial abdominal images at the level of the diaphragmatic crura. To obtain mean T1 values in muscles, an experienced radiologist (OAB) manually drew ROIs around the outline of the diaphragmatic crura, abdominal muscles, and lumbar paraspinal muscles using OsiriX MD (Pixmeo SARL, Geneva, Switzerland), which automatically generated mean T1 values for further analysis.

### 4.3 U‐Net Supported Automatic Segmentation of RT‐MRI Data

*Segmentation*: Image segmentation of RT‐MRI data was performed with a U‐Net [24], a CNN well established in biomedical image segmentation. Here, we developed a combined network that generates separate outputs for the segmentation of lung and diaphragm. From every image plane and patient, five images were manually segmented as training data. To expand the size of the training dataset, we used data augmentation techniques, for example, applying different distortions to generate more training images. The model was implemented with PyTorch [34], and training was performed on an Nvidia GTX 1080 Ti (Nvidia Corp., Santa Clara, USA) using an Adam optimizer with Weight Decay [35] to avoid overfitting. Dice coefficients for the different segmentations performed are provided in Figure S4. See Note S1 for details on the training procedure.

*Post‐Processing*: The diaphragm segmentation yields an image marking the diaphragm as a fuzzy line. To automatically obtain the reference positions for the diaphragm, the center of the line was determined as the value with the highest intensity along the y‐axis chosen for each given x‐position with values greater than the background cut‐off, resulting in a height function of the diaphragm. This was subsequently used for the definition of landmarks and computation of quantities as the area change.

The whole processing and evaluation pipeline was implemented in Python [36] using the packages numpy [37], scipy [38], opencv2 [39], PyTorch [34], and matplotlib [40].

### 4.4 Comparative Examinations

*Pulmonary function tests* were performed according to ATS/ERS standards [4, 41] with a body plethysmography in an upright seated position and a spirometry in supine position (Devices: Vyntus‐Spiro and Master‐Screen‐Body, both CareFusion Germany 234). Maximal inspiratory pressure (MIP) was measured during upright body plethysmography. At least three consecutive tests were performed for all respiratory maneuvers, and the best result was used for further analysis.

*Diaphragm ultrasound* was carried out by a trained pulmonologist (UO) for different breathing maneuvers in supine and upright position. Based on previously described protocols [42, 43], the probe was placed in the intercostal space along the antero‐ and mid‐axillary lines and diaphragm excursion was measured in mm using M‐mode. The device used was a EUP‐5500HV with a EUP‐S50 transducer (both Hitachi Medical Systems).

*Capillary blood gas test* was performed by collecting blood from the arterialized earlobe (Device: GEM‐300, Instrumentation Laboratory). Daytime hypercapnia was defined as pCO_2_ above 45 mmHg [44].

### 4.5 Statistics

Mann–Whitney *U* tests were performed to assess differences in study outcomes between patients and controls. MRI outcomes were not corrected for multiple testing and the significance level was set at *p* ≤ 0.05. Correlation analysis was performed with Pearson or Spearman's correlation coefficient (*r*). The statistical analyses were performed using the software GraphPad Prism 9. Cohen's *d* and its 95% confidence interval for measuring the effect size of variables were calculated using MATLAB R2022b.

## Author Contributions

Rachel Zeng, Omar Al‐Bourini, Ulrike Olgemöller, Sabine Hofer, Matthias Boentert, Ali Seif Amir Hosseini, and Jens Schmidt were responsible for study concept and design. Leonie Lettermann, Leon Lettermann, Tim Friede, Manuel Nietert, Dirk Voit, Jens Frahm, and Martin Uecker contributed to methodology. Leonie Lettermann and Leon Lettermann were responsible for code implementation. Rachel Zeng, Leonie Lettermann, and Matthias Boentert conducted patient recruitment. Rachel Zeng, Omar Al‐Bourini, Leonie Lettermann, and Ulrike Olgemöller conducted investigations. Rachel Zeng and Leonie Lettermann were responsible for data collection and data analysis. Rachel Zeng and Jens Schmidt acquired funding. Rachel Zeng, Leonie Lettermann, and Leon Lettermann were responsible for writing the original draft. All authors were involved in revising the manuscript. All authors have read and approved the final manuscript.

## Funding

This study was supported by the German patient support group for muscle disorders (DGM) (application number Sc21/2) and the German Research Foundation (DFG) within the Clinician Scientist Program “Cell Dynamics in Disease and Therapy” at the University Medical Center Göttingen (project number 413501650).

## Ethics Statement

The study protocol was in accord with good clinical practice and approved by the Medical Ethics Committee at the University Medical Center Göttingen (amendment 1 to protocol 13/10/18). All participants provided written informed consent prior to examination.

## Conflicts of Interest

Jens Frahm and Martin Uecker are co‐inventors and patent holders of the software describing the real‐time MRI technique used here. The remaining authors have no conflicts of interest.

## Supporting information




**Supporting Movie 1**: Movie of RT‐MRI assessment of diaphragmatic motion and chest wall motion in a healthy control.

**Supporting Movie 2**: Movie of RT‐MRI assessment of diaphragmatic motion and chest wall motion in a Pompe patient.

**Supporting Movie 3**: Movie of RT‐MRI assessment of diaphragmatic motion and chest wall motion in a Pompe patient with severe diaphragm weakness.

**Supporting Movie 4**: Movie of RT‐MRI assessment of diaphragmatic motion and chest wall motion in a healthy control while performing the Sniff maneuver.

**Supporting Movie 5**: Movie of RT‐MRI assessment of diaphragmatic motion and chest wall motion in a Pompe patient while performing the Sniff maneuver.

**Supplementary Note 1**: Details of U‐Net training.
**Supporting Figure 1**: Scatter plots of the outcome measures from manual analysis of RT‐MRI assessment of breathing in comparison between Pompe patients and controls.
**Supporting Figure 2**: Scatter plots of all outcome measures from U‐Net based automatic analysis of RT‐MRI assessment of breathing in comparison between Pompe patients and controls.
**Supporting Figure 3**: ROC‐curves of different outcome measures to discriminate between Pompe patients and controls.
**Supporting Figure 4**: DICE scores for U‐Net based segmentation of lung area and diaphragm.
**Supporting Table 1**: Demographics and clinical characteristics of all study participants.
**Supporting Table 2**: Results of Diaphragm Ultrasound of all study participants.
**Supporting Table 3**: Results of T1 mapping of all study participants.

## Data Availability

The program code used for data preparation, training of the network, and analysis is available in the following repository: https://gitlab.gwdg.de/leon.lettermann1/rtmri_lung_unet. An example dataset and the learned weights for the network used in this study are available on zenodo: https://doi.org/10.5281/zenodo.13483534. Additional data supporting the findings of this study are available from the corresponding author upon reasonable request.
