# Supplementary material for: Real‐Time MRI With Deep Learning for Efficient Evaluation of Neuromuscular Breathing Impairment
Source: MedComm (2020). 2026 Feb 24;7(3):e70579. doi: 10.1002/mco2.70579 (PMC12932971; doi:10.1002/mco2.70579)
Supplement: Supplementary file 6 — Supplementary Note 1: Details of U‐Net training. Supporting Figure 1: Scatter plots of the outcome measures from manual analysis of RT‐MRI assessment of breathing in comparison between Pompe patients and controls. Supporting Figure 2: Scatter plots of all outcome measures from U‐Net based automatic analysis of RT‐MRI assessment of breathing in comparison between Pompe patients and controls. Supporting Figure 3: ROC‐curves of different outcome measures to discriminate between Pompe patients and controls. Supporting Figure 4: DICE scores for U‐Net based segmentation of lung area and diaphragm. Supporting Table 1: Demographics and clinical characteristics of all study participants. Supporting Table 2: Results of Diaphragm Ultrasound of all study participants. Supporting Table 3: Results of T1 mapping of all study participants. [file MCO2-7-e70579-s002.pdf]

Zeng, Al-Bourini, Lettermann et al.

Real-time MRI with deep learning for efficient evaluation of neuromuscular breathing impairment

Supplementary Material

Table of contents

Supplementary Note 1 – Details of U-Net training. 2

Figure S1. Scatter plots of the outcome measures from manual analysis of RT-MRI assessment of breathing in comparison between Pompe patients and controls 3

Figure S2. Scatter plots of all outcome measures from U-Net based automatic analysis of RT-MRI assessment of breathing in comparison between Pompe patients and controls. 5

Figure S3. ROC-curves of different outcome measures to discriminate between Pompe patients and controls. 6

Figure S4. DICE scores for U-Net based segmentation of lung area and diaphragm. 7

Table S1. Demographics and clinical characteristics of all study participants. 8

Table S2. Results of Diaphragm Ultrasound of all study participants. 10

Table S3. Results of T1 mapping of all study participants. 11

Movie S1: Movie of RT-MRI assessment of diaphragmatic motion and chest wall motion in a healthy control. 12

Movie S2: Movie of RT-MRI assessment of diaphragmatic motion and chest wall motion in a Pompe patient. 12

Movie S3: Movie of RT-MRI assessment of diaphragmatic motion and chest wall motion in a Pompe patient with severe diaphragm weakness. 12

Movie S4: Movie of RT-MRI assessment of diaphragmatic motion and chest wall motion in a healthy control while performing the Sniff maneuver. 12

Movie S5: Movie of RT-MRI assessment of diaphragmatic motion and chest wall motion in a Pompe patient while performing the Sniff maneuver. 12

Supplementary References 12

Supplementary Note 1 – Details of U-Net training.

The U-Nets were trained based on prepared segmentations drawn by hand by LL with verification by OAB. As the coronal planes had two lungs to differentiate (left-right), and sagittal planes had a slightly different resolution, independent networks were trained for the segmentation of coronal and sagittal sequences.

For the coronal network, 334 masks were drawn. Data augmentation generated 19 additional pairs per hand drawn mask, yielding 6080 masks used for training and 600 masks for validation, where the training/validation split was performed ahead of data augmentation. Manual validation was performed by RZ, OAB and LL on the full segmentation of the videos, containing 200 frames per sequence.

The sagittal network analogously used 225 hand drawn masks before augmentation, split into 4100 training and 400 validation images.

Both networks were trained on a Nvidia GeForce GTX 1080 Ti, using a cross entropy loss for the area segmentation and a binary cross entropy loss for the diaphragm. Training was performed for 100 epochs with 100 training images per mini batch, using an AdamW optimizer with an initial learning rate of 1e-3, a weight decay of 3e-1, and an exponential learning rate decay factor of 0.99.

Figure S1

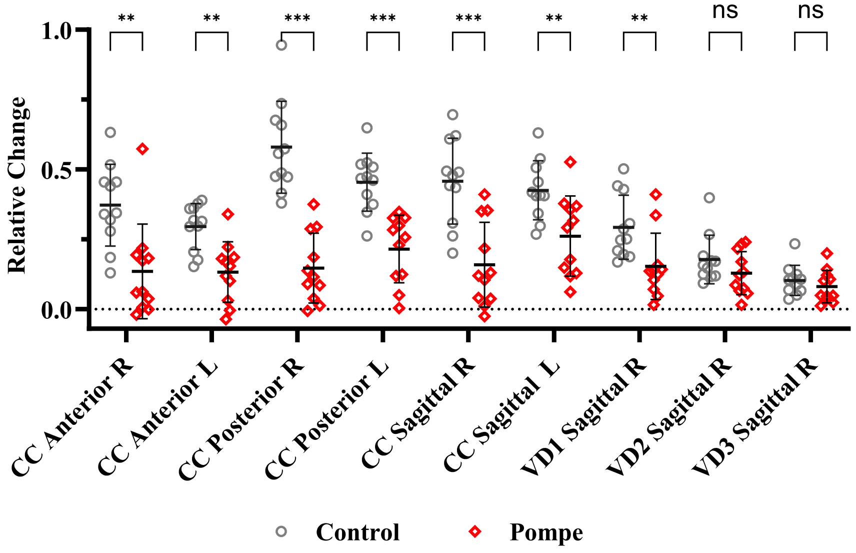

Figure S1. Scatter plots of the outcome measures from manual analysis of RT-MRI assessment of breathing in comparison between Pompe patients and controls. CC, cranio-caudal height of lung; VD ventro-dorsal excursion of chest wall; R: right hemithorax; L: left hemithorax. Significance levels are reported as "*" for $p < 0.05$, "**" for $p < 0.01$, "***" for $p < 0.001$ and "ns" for not significant.

Figure S2

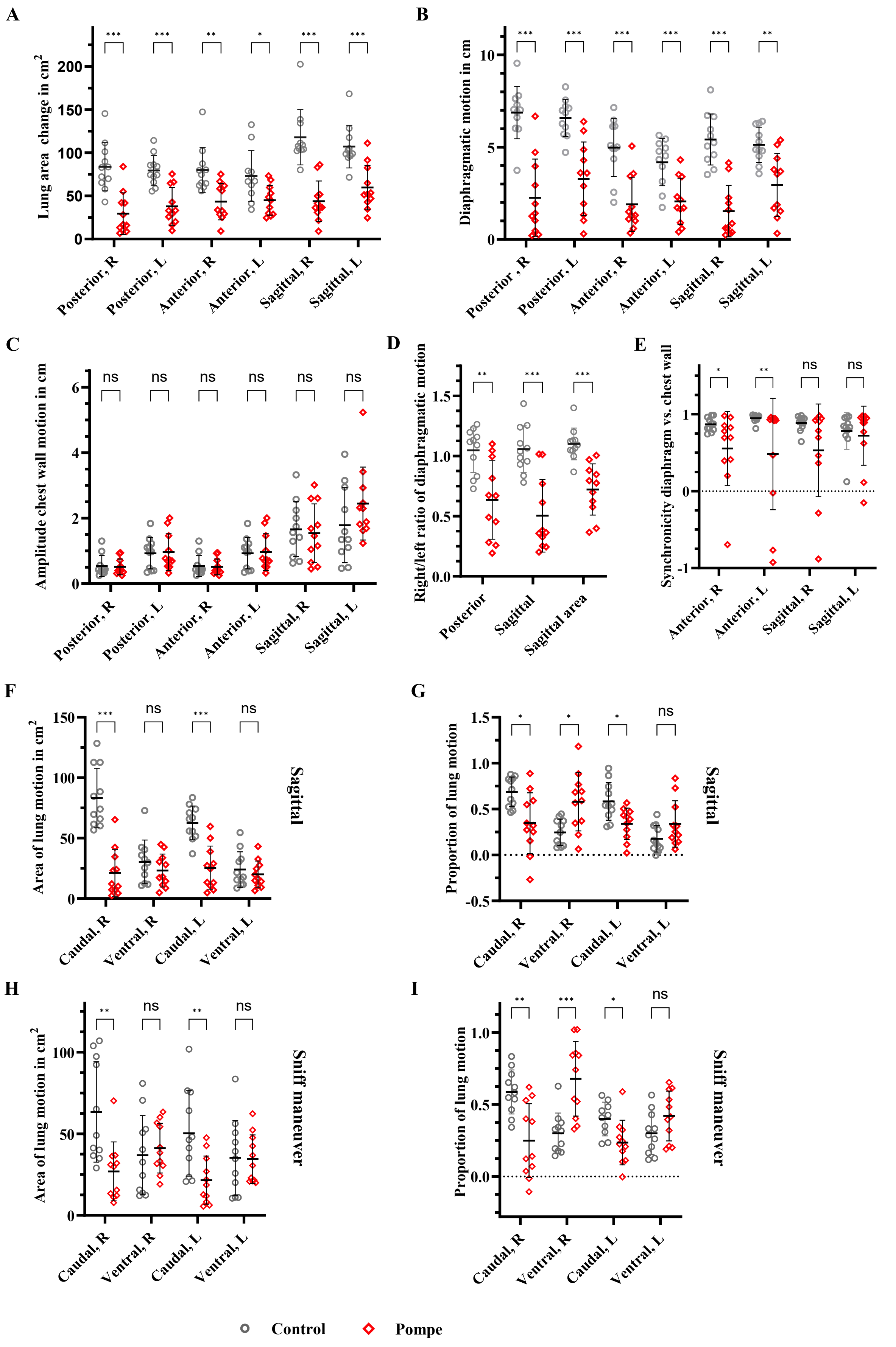
A
Lung area change in cm2
Posterior, R
Posterior, L
Anterior, R
Anterior, L
Sagittal, R
Sagittal, L
B
Diaphragmatic motion in cm
C
Amplitude chest wall motion in cm
ns
D
Right/left ratio of diaphragmatic motion
Posterior
Sagittal
Sagittal area
E
Synchronicity diaphragm vs. chest wall
F
Area of lung motion in cm2
Caudal, R
Ventral, R
Caudal, L
Ventral, L
Sagittal
G
Proportion of lung motion
H
Sniff maneuver
I
Control
Pompe

Figure S2. (previous page) Scatter plots of all outcome measures from U-Net based automatic analysis of RT-MRI assessment of breathing in comparison between Pompe patients and controls. Pompe patients showed significantly reduced total lung area change (A) and diaphragmatic motion (B) during deep breathing compared to controls, while chest wall motion (C) did not differ significantly between the two cohorts. Pompe patients showed an asymmetric pattern with the right hemidiaphragm more affected than the left hemidiaphragm (D) and less synchronicity between diaphragm and chest wall motion compared to controls (E) (+1 indicates complete synchronicity with diaphragm and chest wall expanding and deflating simultaneously and -1 indicates complete asynchronicity with diaphragm and chest wall moving oppositely). Analysis of the separate contributions of diaphragm and chest wall motion to total lung change demonstrated that the diaphragm is the main component of respiratory motion in the control group, whereas in LOPD patients the contribution of the chest wall was relatively greater during deep breathing (F+G) and even increased slightly during a voluntary sniff maneuver (H+I), reflecting additional chest expansion to compensate for diaphragm weakness. Anterior, anterior imaging plane; Posterior, posterior imaging plane; Sagittal, sagittal imaging plane; Caudal, diaphragm-related outcomes; Ventral, chest wall-related outcomes; R, right hemithorax; L, left hemithorax. Significance levels as in Fig. S1.

Figure S3

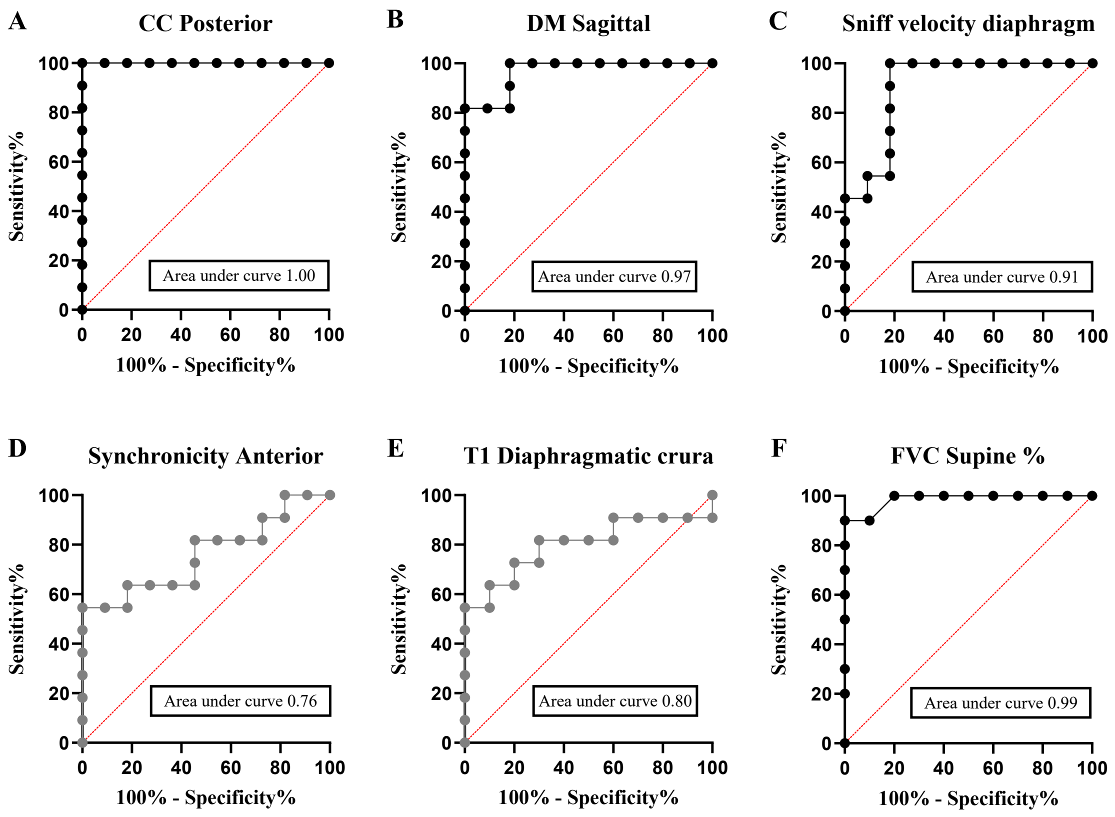

Figure S3. ROC-curves of different outcome measures to discriminate between Pompe patients and controls.

ROC-curves of sensitivity and specificity for differentiating between Pompe patients and controls based on RT-MRI of diaphragmatic excursion using manual analysis (A) and U-NET supported automatic analysis (B), RT-MRI of diaphragmatic sniff velocity (C) and synchronicity between diaphragm and chest wall (D), T1 mapping of the diaphragmatic crura (E), and FVC measured in supine position (F). For better overview, only results from the right hemithorax are shown for RT-MRI outcomes. CC, cranio-caudal change of lung size; DM, diaphragmatic motion; Anterior, anterior imaging plane; Posterior, posterior imaging plane; Sagittal, sagittal imaging plane; FVC, forced vital capacity.

Figure S4

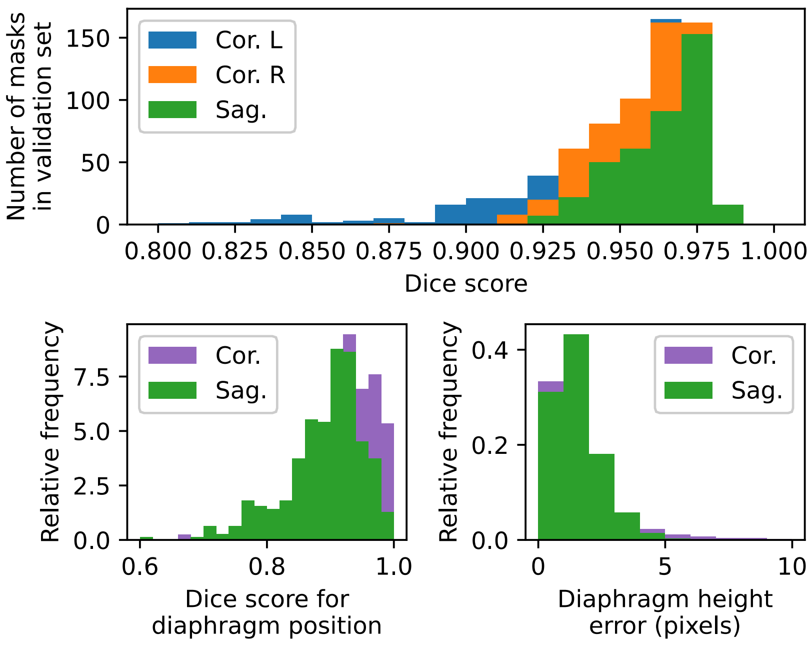

Figure S4. DICE scores for U-Net based segmentation of lung area and diaphragm. A: Dice-Sørensen coefficient[S1,S2] for the 2D segmentation of the lung area, compared between left and right lung in coronal view, and sagittal views. **B:** Dice-Sørensen coefficients for the 1D segmentation of the diaphragm position (in the left-right axis for coronal, or dorsal-ventral axis sagittal). **C:** Error of the diaphragm height obtained from the diaphragm segmentation.

Table S1. Demographics and clinical characteristics of all study participants.

Participant	Gender	BMI, (kg/m^2)	Age range (years)	Disease duration (years)	Symptoms of dyspnoea (mMRC scale)	Muscle sum score (modified MRC scale)	R-Pact (Pompe-specific activity scale)	Duration of ERT	NIV	FVC upright (% of predicted)	FVC supine (% of predicted)	P0,1 (% of predicted)	MIP (kPa)	pCO_2 (mmHg)
C01	f	25	45-55	NA	0	139	NA	NA	NA	100	97	194	7.5	37
C02	f	20	25-35	NA	0	140	NA	NA	NA	84	82	151	2.71	33
C03	m	23	60-70	NA	0	134	NA	NA	NA	78	72	183	4.76	*NA
C04	m	24	35-45	NA	1	140	NA	NA	NA	103	91	94	12.37	36
C05	m	28	50-60	NA	0	140	NA	NA	NA	89	89	190	7.79	38
C06	f	21	20-30	NA	0	140	NA	NA	NA	91	86	102	3.79	*NA
C07	m	31	70-80	NA	1	140	NA	NA	NA	98	105	239	6.7	33
C08	m	26	55-65	NA	0	133.25	NA	NA	NA	95	94	177	7.42	33
C09	f	33	45-55	NA	1	140	NA	NA	NA	75	84	248	6.69	33
C10	f	32	50-60	NA	0	140	NA	NA	NA	108	102	193	11.72	35
C11	m	25	50-60	NA	0	140	NA	NA	NA	99	92	233	*NA	38
P01	f	22	45-55	13	1	135	23	11 years	no	94	67	141	5.11	35
P02	f	20	30-40	4	0	129	21	6 months	no	68	47	81	3.43	38
P03	m	20	55-65	8	2	132	21	3 years	BiPAP	63	63	94	3.46	*NA
P04	m	25	45-55	7	1	138	30	2 months	no	75	56	180	5.1	36
P05	m	23	55-65	28	1	138.75	27	5 years	BiPAP	78	48	58	5.8	45
P06	f	20	20-30	9	0	136.75	29	9 years	no	87	82	72	0.65	45
P07	m	25	65-75	15	2	133.25	16	discontinued	BiPAP	64	47	131	3.31	37
P08	m	27	55-65	14	1	137.5	28	6 years	CPAP	65	66	65	8.53	38
P09	f	20	55-65	28	1	129.25	21	15 years	BiPAP	86	56	64	4.83	39
P10	f	21	55-65	45	1	107.5	18	discontinued	no	80	64	238	4.03	39
P11	m	26	50-60	3	1	135	31	4 months	no	55	40	132	9.55	41

Table S1. (previous page). The MRC scale (Medical Research Council scale) was used for evaluation of muscle strength (0 to 5) [S3], including head flexion/extension, arm abduction, elbow flexion/ extension, wrist flexion/extension, finger flexion/extension, hip flexion/extension, knee flexion/extension, and ankle flexion/ extension. Rating of both sides led to a maximum MRC sum score of 140. The mMRC (modified Medical Research Council) Dyspnea Scale was used to assess the patient-reported breathlessness during daily activities. The scale ranges from 0 points (breathlessness only during strenuous exercise) to 4 points (too breathless to leave the house) [S4,S5]. The patient-based Rasch-built Pompe-specific activity (R-PAct) scale [S6] was used to assess Pompe patient´s ability to carry out daily activities. A maximum of 36 points (no impairment in daily activity) could be reached.

NA, not applicable; *NA, data not available for organizational reasons, e.g. device defect.

Table S2. Results of Diaphragm Ultrasound of all study participants.

Participant	Ultrasound deep breathing seated right (mm)	Ultrasound deep breathing supine right (mm)	Ultrasound tidal breathing seated right (mm)	Ultrasound tidal breathing supine right (mm)	Ultrasound sniff seated right (mm)	Ultrasound sniff supine right (mm)	Ultrasound deep breathing seated left (mm)	Ultrasound deep breathing supine left (mm)	Ultrasound tidal breathing seated left (mm)	Ultrasound tidal breathing supine left (mm)	Ultrasound sniff seated left (mm)	Ultrasound sniff supine left (mm)
C01	62.3	52.7	13.1	10.5	36.5	62.2	35	49.2	32.2	19.6	44.6	48.9
C02	38.8	65.7	33.8	21.1	38.1	43.2	32.7	49.8	31.6	13.5	47.9	37.5
C03	44.4	39.3	35.4	28.7	46.1	30.9	32.5	36.3	22.4	18.1	40.3	27.2
C04	65.2	40.9	17.4	17.4	52.4	44.9	34.7	37.4	14.7	11.6	28.2	26.7
C05	31.5	42.3	16.5	18.5	27.5	27.4	49	44.5	15.1	19.1	31.1	28.3
C06	30.5	32.3	24.8	22.6	31	28.5	46.2	28.7	34.2	23.4	44.6	25
C07	28.6	26.4	16.5	18.3	21.2	22.4	26.8	42.1	17.2	18.3	19.5	24.7
C08	46.7	35.4	20.9	23.6	47.2	64.4	50.7	54.8	33.4	30.6	62.3	55.3
C09	44.3	31.2	24.2	22.4	41.8	27.6	47.2	43.6	26.7	19.2	40.9	36.3
C10	45.6	45.4	34.4	21.8	38.1	37.1	§NA	§NA	§NA	§NA	§NA	§NA
C11	54	49	21	7	56	44	62	49	14	8	64	53
P01	28.2	26.5	19.2	18.6	19.6	18.2	26.3	20.4	17.6	14.2	22.3	16.8
P02	23.6	24.3	13.8	20.3	18.8	19.1	22.2	23.4	15.6	14.2	18.8	24.6
P03	15.6	16.5	12.8	11.2	19.6	19.6	16.7	17.2	13.1	12.4	18.9	19.8
P04	30.1	32.5	21.4	20.9	34.2	36.2	38.4	44.9	17.2	19.2	35.4	41.2
P05	26.8	15.7	22.7	10.6	29.2	13.1	39.6	16.9	22.4	12.4	28.4	14.7
P06	19.2	12.4	15.6	11.6	12.3	9.8	21.6	10.8	19.2	10.7	17.9	9.7
P07	26.3	22.8	12.5	11.9	19	17.6	23.7	17.3	15.4	12.1	16.3	16.9
P08	18.3	36.4	19.5	23.2	25.1	33.4	21.6	44.3	18.7	22.8	25.1	36.8
P09	28	21.6	13.4	16.5	14.7	20.7	27.1	19.6	11.6	11.6	37.4	22.3
P10	36.7	25	23.8	14.2	32.3	23.6	33.4	24.3	26.9	11.3	43.9	33.4
P11	16.1	20	6.7	12.2	15.6	24.4	§NA	§NA	§NA	§NA	§NA	§NA

§NA, data not available as the left hemidiaphragm couldn't be visualised sonographically due to anatomical limitations.

Table S3. Results of T1 mapping of all study participants.

Participant	Diaphragmatic crura right (ms)	Diaphragmatic crura left (ms)	Lumbar paraspinal muscles (ms)	Abdominal muscles (ms)
C01	1141.91	1220.30	1155.55	1239.75
C02	1268.74	1359.25	1179.62	1099.94
C03	600.60	934.32	1155.63	1282.43
C04	1152.96	814.89	1160.31	1296.45
C05	1029.12	1080.42	1128.95	1245.52
C06	1340.87	1391.96	1160.10	1137.37
C07	#NA	#NA	#NA	#NA
C08	1110.39	1014.62	1250.48	1290.42
C09	923.67	546.09	1098.03	1284.47
C10	1179.37	1135.86	1128.96	732.66
C11	1201.05	1146.07	1160.91	1242.60
P01	967.72	397.23	1434.40	1327.55
P02	771.04	205.57	1344.16	1474.47
P03	185.23	222.90	1286.07	1042.16
P04	83.98	82.16	597.44	270.59
P05	116.11	200.86	705.93	448.28
P06	1583.02	1574.52	1217.25	1236.92
P07	132.64	161.48	757.63	769.84
P08	81.39	89.00	379.46	693.05
P09	257.13	294.81	611.61	798.52
P10	1167.12	277.71	706.39	1044.88
P11	1101.79	952.23	320.86	332.38

#NA, data not available due to imaging artefacts.

Movie S1: Movie of RT-MRI assessment of diaphragmatic motion and chest wall motion in a healthy control. The left and right hemidiaphragms demonstrate synchronous displacement during deep breathing; chest wall excursion is minimal. a) The raw MRI image, recorded in a coronal plane. b) MRI image with overlay of the segmentation of the lung (yellow, red borders) and diaphragm segmentation (green). c) The segmentation masks. d) The MRI image overlain with the section over which the chest wall motion is computed. The time series in the right panels show the dynamics of the diaphragmatic motion (e, green), the chest wall motion (f, orange) and the total lung area change (g, blue), for left (full) and right (dashed) lung respectively.

Movie S2: Movie of RT-MRI assessment of diaphragmatic motion and chest wall motion in a Pompe patient. The right hemidiaphragm demonstrates minimal excursion, whereas the left shows relatively greater excursion during deep breathing. Chest wall motion is not limited. a) The raw MRI image, recorded in a coronal plane. b) MRI image with overlay of the segmentation of the lung (yellow, red borders) and diaphragm segmentation (green). c) The segmentation masks. d) The MRI image overlain with the section over which the chest wall motion is computed. The time series in the right panels show the dynamics of the diaphragmatic motion (e, green), the chest wall motion (f, orange) and the total lung area change (g, blue), for left (full) and right (dashed) lung respectively.

Movie S3: Movie of RT-MRI assessment of diaphragmatic motion and chest wall motion in a Pompe patient with severe diaphragm weakness. Nearly no diaphragmatic motion can be observed. Chest wall motion is preserved. a) The raw MRI image, recorded in a coronal plane. b) MRI image with overlay of the segmentation of the lung (yellow, red borders) and diaphragm segmentation (green). c) The segmentation masks. d) The MRI image overlain with the section over which the chest wall motion is computed. The time series in the right panels show the dynamics of the diaphragmatic motion (e, green), the chest wall motion (f, orange) and the total lung area change (g, blue), for left (full) and right (dashed) lung respectively.

Movie S4: Movie of RT-MRI assessment of diaphragmatic motion and chest wall motion in a healthy control while performing the Sniff maneuver. Sniff is initiated by caudal displacement of the diaphragm, followed by ventral chest wall excursion. Diaphragmatic motion contributes more than chest wall motion to the overall area change. a) The raw MRI image, recorded in a sagittal plane. b) MRI image with overlay of the segmentation of the lung (yellow, red borders) and diaphragm segmentation (green). c) The segmentation masks. d) The MRI image overlain with the section over which the Chest Wall motion is computed. The time series in the right panels show the dynamics of the diaphragmatic motion (e, green), the chest wall motion (f, orange) and the lung area (g). The lung area is separated into the total area (blue), the area as caused by the diaphragmatic motion (green), and the area as caused by chest wall motion (orange).

Movie S5: Movie of RT-MRI assessment of diaphragmatic motion and chest wall motion in a Pompe patient while performing the Sniff maneuver. Sniff maneuver is initiated by ventral chest wall excursion accompanied by paradoxical upward diaphragmatic displacement. a) The raw MRI image, recorded in a sagittal plane. b) MRI image with overlay of the segmentation of the lung (yellow, red borders) and diaphragm segmentation (green). c) The segmentation masks. d) The MRI image overlain with the section over which the Chest Wall motion is computed. The time series in the right panels show the dynamics of the diaphragmatic motion (e, green), the chest wall motion (f, orange) and the lung area (g). The lung area is separated into the total area (blue), the area as caused by the diaphragmatic motion (green), and the area as caused by chest wall motion (orange).

Supplementary References

S1. Sørensen T. A method of establishing groups of equal amplitude in plant sociology based on similarity of species and its application to analyses of the vegetation on Danish commons. *Biol Skrifter / Kongelige Danske Videnskabernes Selskab*. 1948.
S2. Dice LR. Measures of the Amount of Ecologic Association Between Species. *Ecology*. 1945;26(3):297-302. doi:10.2307/1932409
S3. Medical Research Council. Aids to examination of the peripheral nervous system. *Memorandum no. 45. London: Her Majesty's Stationary Office*. 1976.
S4. Mahler DA, Wells CK. Evaluation of clinical methods for rating dyspnea. *Chest*. 1988;93(3):580-586. doi:10.1378/chest.93.3.580
S5. Hajiro T, Nishimura K, Tsukino M, Ikeda A, Koyama H, Izumi T. Analysis of clinical methods used to evaluate dyspnea in patients with chronic obstructive pulmonary disease. *Am J Respir Crit Care Med*. 1998;158(4):1185-1189. doi:10.1164/ajrccm.158.4.9802091
S6. van der Beek NAME, Hagemans MLC, van der Ploeg AT, van Doorn PA, Merkies ISJ. The Rasch-built Pompe-specific activity (R-PAct) scale. *Neuromuscul Disord*. 2013;23(3):256-264. doi:10.1016/j.nmd.2012.10.024
